# Comparative Analysis of Muscle Fibers in Selected Muscles of Working and Companion Dog Breeds

**DOI:** 10.3390/ani14243576

**Published:** 2024-12-11

**Authors:** Cezary Osiak-Wicha, Katarzyna Kras, Marcin B. Arciszewski

**Affiliations:** Department of Animal Anatomy and Histology, Faculty of Veterinary Medicine, University of Life Sciences in Lublin, Akademicka 12, 20-950 Lublin, Poland; katarzyna.kras@up.lublin.pl (K.K.); mb.arciszewski@wp.pl (M.B.A.)

**Keywords:** MYH2, MYH4, MYH7, triceps brachii, biceps femoris

## Abstract

The structural and functional characteristics of skeletal muscle fibers play a crucial role in understanding the physical capabilities of dogs, particularly in relation to their breed-specific roles. This study aimed to compare the muscle fiber composition of working and companion dog breeds by analyzing the triceps brachii and biceps femoris muscles, focusing on fiber morphology, myosin heavy chain (MYH) isoform distribution, and nuclei per fiber. A total of 12 dogs, divided equally into working and companion breed groups, were used in this study. Muscle samples were collected post-mortem and prepared for histological analysis using cryosectioning. Immunohistochemical staining was employed to identify the expression of MYH isoforms, including MYH2, MYH4, and MYH7, which correspond to type IIa, IIb, and type I fibers, respectively. The results demonstrated significant differences between the two breed groups. Working dogs exhibited larger muscle fibers, a higher proportion of type IIa (MYH2) and type I (MYH7) fibers, and a greater number of nuclei per fiber, suggesting adaptations for endurance and strength. In contrast, companion dogs showed a higher proportion of type IIb (MYH4) fibers, indicative of their capacity for short bursts of activity rather than sustained exertion. Companion breeds also displayed a higher fiber density but fewer nuclei per fiber, which may contribute to slower muscle regeneration. These findings may provide insights into the muscle adaptations of dogs based on their breed-specific functional demands and highlight the importance of considering these differences in veterinary care and rehabilitation. The study underscores the influence of selective breeding on muscle structure and function in dogs and suggests further research into breed-specific muscle recovery mechanisms.

## Simple Summary

This study compared the muscle fiber composition of working and companion dog breeds to understand how their muscles adapt to breed-specific tasks. By analyzing the triceps brachii and biceps femoris muscles of 12 dogs, we found that working dogs had larger muscle fibers, a higher proportion of endurance-related fibers (types IIa and I), and more nuclei per fiber, which support strength and endurance. Companion dogs, on the other hand, had more fibers suited for short bursts of activity (type IIb) and fewer nuclei per fiber, suggesting slower muscle regeneration. These results highlight the role of selective breeding in shaping muscle structure and function in dogs.

## 1. Introduction

The structure and function of skeletal muscle fibers represent a crucial aspect of understanding the physiological capabilities of dogs, particularly in relation to their breed-specific roles [1,2]. Working dog breeds, such as those used in herding, hunting, or guarding, demonstrate unique muscle fiber characteristics that enable them to perform tasks requiring endurance, strength, and speed [3,4,5]. In contrast, companion dog breeds, primarily bred for temperament and companionship, may exhibit different muscular adaptations, particularly in muscles related to posture [6,7], as these traits align with their less physically demanding lifestyles. This comparative analysis focuses on two specific muscles, the triceps brachii and biceps femoris, which are essential for locomotion in both groups yet likely exhibit significant morphological and functional differences between working and companion breeds.

Muscle fibers can be classified based on the expression of specific myosin heavy chain (MYH) isoforms, which are closely associated with the fibers’ contractile and metabolic properties. MYH2 is predominantly expressed in type IIa fibers, which are fast-twitch fibers with both oxidative and glycolytic capacities [8]. These fibers are versatile, supporting both sustained activities and short bursts of power. MYH4 is associated with type IIb fibers, which are fast-twitch, glycolytic fibers specialized for rapid, powerful contractions but fatigue quickly due to their reliance on anaerobic metabolism. These fibers are well-suited for activities that require short, intense bursts of strength. MYH7, on the other hand, is expressed in type I fibers, which are slow-twitch, oxidative fibers [9,10,11]. These fibers are highly resistant to fatigue and are optimized for endurance activities, relying on aerobic metabolism to sustain prolonged, low-intensity contractions. Together, these different fiber types contribute to the functional specialization of muscles, allowing for a range of activities from endurance to powerful, rapid movements [12].

The triceps brachii and biceps femoris are two critical muscles involved in the movement of the forelimbs and hindlimbs, respectively. The triceps brachii, located in the upper forelimb, is primarily responsible for elbow extension and plays a pivotal role in maintaining posture, since in quadrupeds such as dogs, the forelimbs account for approximately 60% of body weight support, making the muscles of the forelimb, including the triceps brachii, crucial for stability and endurance [13,14]. The biceps femoris, found in the posterior hindlimb, facilitates the extension of the hip and flexion of the knee and is essential for propulsion and activities such as jumping, running, or sprinting. Both muscles are composed of various types of muscle fibers, including slow-twitch (type I) and fast-twitch (type II) fibers, which contribute to their functional properties [15]. Slow-twitch fibers are associated with endurance activities and are more prevalent in muscles requiring sustained contraction, while fast-twitch fibers are responsible for short bursts of speed or strength [9,16,17,18].

Despite the wealth of information on macroscopic muscle anatomy, there remains a gap in understanding the microstructural adaptations of muscles at the fiber level, particularly when comparing working and companion breeds. Studies such as those by Guy and Snow (1981) [19] have laid detailed groundwork for this understanding by examining muscle fiber morphology and myosin isoform distribution in different dog breeds. However, these studies did not explore the differences between breed types in detail, leaving unanswered questions about how specific muscle fibers contribute to the functional variations observed among different breed groups [19]. This study seeks to fill this gap by providing a detailed analysis of the triceps brachii and biceps femoris muscles in both working and companion dog breeds, with a focus on the morphology of muscle fibers and myosin isoform distribution.

We hypothesize that working breeds, having been selectively bred for physically demanding tasks, will exhibit muscle fiber compositions that enhance their ability to perform powerful, rapid movements and sustain physical endurance for activities such as herding and hunting. In contrast, companion breeds, typically bred for traits that do not prioritize physical exertion, are expected to show a muscle fiber composition that reflects their comparatively lower physical demands. These adaptations likely influence differences in muscle function, with working breeds being better suited for tasks requiring strength and endurance, while companion breeds may have a muscle composition more tailored to brief, low-intensity activities and postural maintenance [20,21].

By investigating muscle fiber composition and myosin isoform distribution, the study seeks to enhance our understanding of how selective breeding and specific functional demands influence the muscle structure of different breeds. This knowledge may provide insights into the relationship between muscular adaptations and breed-specific roles, contributing to fields such as veterinary medicine, animal breeding, and canine rehabilitation. For example, breeds used for physically demanding roles such as herding or search-and-rescue could be selectively bred to enhance traits favoring slower-twitch, endurance-oriented fibers, promoting muscle health and longevity, while companion breeds might benefit from fast-twitch muscle fibers, optimizing them for short bursts of intense activity. In rehabilitation, these insights may be equally valuable, as they allow veterinarians and physiotherapists to design breed-specific recovery protocols that align with a dog’s natural muscle composition. For instance, dogs with a higher proportion of fast-twitch fibers might benefit from targeted, low-intensity exercises to maintain muscle function without overstressing their rapid-contracting fibers, which are prone to fatigue.

## 2. Materials and Methods

### 2.1. Animals and Samples Collection

The study was performed in compliance with national animal protection regulations (Animal Experimentation Act, dated 15 January 2015), which align with European legislation on animal experiment ethics (Directive 2010/63/EU). The study was conducted using two distinct groups of dogs, categorized as either working or companion breeds. In the working group, only breeds that have historically been bred for physical tasks, such as herding, guarding, or hunting, were included (such as German Shepard or Doberman Pinscher). Dogs used in the study, while belonging to working breeds, were not actual actively working dogs. Dogs were classified into the working group based on the Fédération Cynologique Internationale (FCI) guidelines, which define working breeds as those eligible for working tests and capable of obtaining a Working Class Certificate (WCC). The companion group consisted of breeds bred exclusively for companionship, such as the Shih Tzu, or breeds that were historically working dogs but are now predominantly kept as companions, such as the Yorkshire Terrier. A total of twelve dogs were used, with six dogs per group (*n* = 6). The dogs were selected based on breed and age to ensure as much uniformity as possible between the two groups (Table 1).

No dogs were harmed or euthanized for the purpose of this study. All muscle samples were collected post-mortem from dogs that died naturally or from unrelated medical conditions, with no known diseases or health conditions that could have impacted the structure or function of their muscles. These samples were obtained from a veterinary clinic following death, ensuring ethical collection practices. Muscle samples were harvested immediately after death from the mid-region of both the triceps brachii and biceps femoris, according to the previously described protocol [22]. Immediately after extraction, the samples were embedded in Tissue Freezing Medium (Leica Biosystems, Nussloch, Germany) and rapidly frozen using 2-methylbutane (Sigma-Aldrich, Darmstadt, Germany) chilled in liquid nitrogen, to preserve tissue integrity. The frozen samples were then transported on ice to the laboratory, where they were stored at −80 °C until further analysis.

### 2.2. Samples Preparation

After collection, the muscle samples from the triceps brachii and biceps femoris were prepared for histological analysis using a cryostat. Each muscle was embedded in Tissue Freezing Medium (Leica Biosystems, Nussloch, Germany) and sectioned using a cryostat (HM 525 NX, Thermo Scientific, Waltham, MA, USA) at a controlled temperature of −20 °C to ensure precise and consistent slicing. To locate the anatomical middle of the muscle belly, sections were cut progressively from one end of the muscle to the other. The thickness of each section was measured, and the section size was recorded as it increased toward the mid-region of the muscle. Once the section size reached its maximum and began to decrease, this was identified as the middle of the muscle belly. For each muscle from each animal, five sections were selected for analysis. These sections were chosen using a systematic random sampling method to ensure unbiased selection. Specifically, every third section from the middle region was chosen until the desired number of five sections was obtained. All sections were cut to a thickness of 10 μm. The prepared sections were subsequently stored at −80 °C until staining and further analysis [22,23].

### 2.3. Immunohistochemistry

The immunohistochemical analysis for detecting MYH2, MYH4, and MYH7 in the analyzed muscles was performed following the protocol previously described by Arbatowska et al. [24], with modifications for frozen tissue sections. Briefly, once the sections reached room temperature, they were rinsed in phosphate-buffered saline (PBS, 0.1 M, pH = 7.3) three times, then endogenous peroxidases were blocked with 3% H_2_O_2_ for 10 min, and nonspecific protein binding sites were blocked using UltraVision Protein Block (Thermo Scientific, Waltham, MA, USA) for 5 min. The primary antibodies (one antibody per section) directed against MYH2, MYH4, and MYH7 (Table 1) diluted in antibody diluent (Emerald, Cell Marque Corp., Rocklin, CA, USA) were then applied, and sections were incubated overnight at 4 °C. A control reaction was conducted simultaneously, where the antibody diluent was applied to the sections instead of primary antibodies. The following day, the sections were incubated with a BrightVision two-step detection system of poly-HRP-anti Ms/Rb IgG (ImmunoLogic WellMed B.V., Duiven, The Netherlands, Table 2) for a total of 45 min, and the antibody complexes were visualized using 3,3′-diamino-benzidine (DAB substrate kit, ab64238, Abcam, Cambridge, UK). The reaction was stopped with distilled water, and the slides were counterstained with Meyer’s hematoxylin (Patho, Mar-Four, Konstantynów Łódzki, Poland). The stained sections were rinsed under running water for 10 min, dehydrated in an ascending alcohol series, cleared in xylene, mounted with Shandon Consul-Mount (Thermo Scientific, Waltham, MA, USA), and dried. Between each step, up to the DAB reaction, slides were rinsed in PBS. Unless otherwise noted, all steps were carried out at room temperature. The sections were examined using a light microscope (BX-51 DSU, Olympus, Tokyo, Japan) equipped with a digital color camera (DP-70, Olympus, Tokyo, Japan). High-resolution digital images were captured using Cell^M 2.3 software (Olympus, Tokyo, Japan) under standardized lighting conditions, with consistent brightness and contrast settings, by a single person. No positive immunoreaction was observed in the control sections.

### 2.4. Morphometric Analysis

The obtained images were analyzed using the ImageJ 1.52 software [25]. The measurements included fascicle area [µm^2^], fiber area [µm^2^], fiber area per fascicle area, fibers per 1000 µm^2^ of fascicle, and nuclei per fiber. Percentages of MYH2-immunoreactive (IR) fibers, MYH4-IR fibers, and MYH7-IR fibers were calculated and expressed as a percentage of the total number of fibers. Fiber distribution was calculated by comparing the percentages of fibers between muscles in each group.

### 2.5. Statistical Analysis

All statistical analyses were conducted using GraphPad Prism version 10.3.0 for Windows (GraphPad Software, San Diego, CA, USA). The normality of the data was assessed using the Shapiro–Wilk test. For data that were normally distributed, an unpaired t-test was performed to compare differences between the working and companion dog groups. For data that did not follow a normal distribution, the non-parametric Mann–Whitney U test was used. Statistical significance was set at *p* < 0.05 for all comparisons. Results are presented as mean ± standard deviation (SD).

## 3. Results

### 3.1. Triceps Brachii

**Figure 1:**
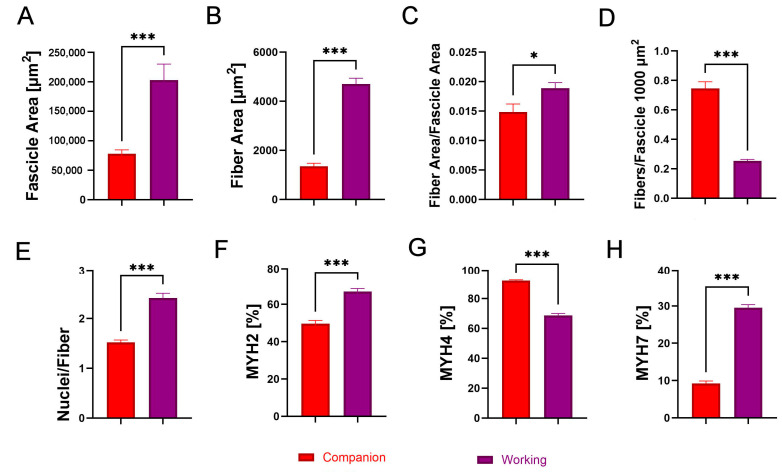
Comparative analysis of (**A**) fascicle area [µm^2^], (**B**) fiber area [µm^2^], (**C**) fiber area per fascicle area, (**D**) fibers per 1000 µm^2^ of fascicle, (**E**) nuclei per fiber, (**F**) percentage of MYH2-immunoreactive (IR) fibers, (**G**) percentage of MYH4-IR fibers, and (**H**) percentage of MYH7-IR fibers in the triceps brachii of companion (red) and working (purple) dogs. Asterisks (*) indicate significant differences between companion and working dogs (* *p* < 0.05, *** *p* < 0.001).

**Figure 2:**
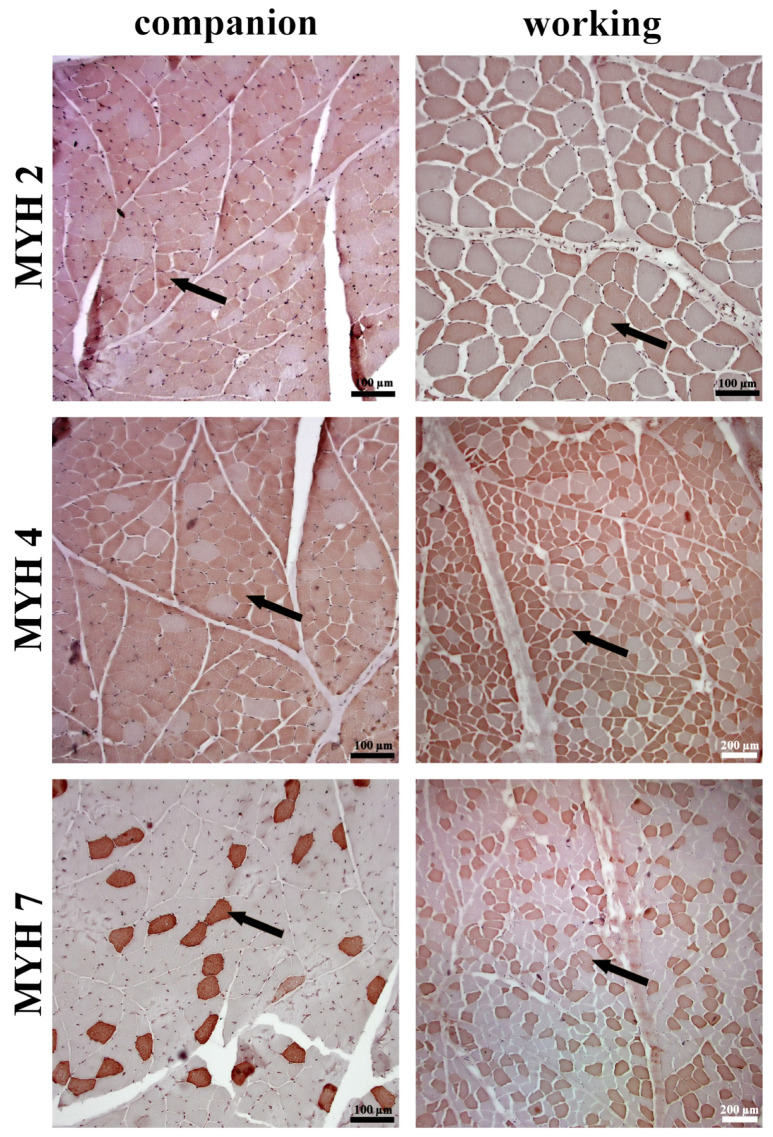
Immunohistochemical staining of muscle fibers in the triceps brachii of companion and working dog breeds, illustrating the distribution of different myosin heavy chain (MYH) isoforms. Staining was performed using DAB as a chromogen. The rows represent different myosin heavy chain isoforms, with MYH2, MYH4, and MYH7 detected in companion and working breeds. Arrows indicate immunoreactive muscle fibers displaying the respective MYH isoform. Scale bars are included in each image for reference (100 µm and 200 µm).

The comparative analysis between companion and working dog breeds revealed significant differences in muscle structure and fiber type composition. In terms of fascicle area, working dogs exhibited a significantly larger fascicle size compared to companion dogs (Figure 1A; *p* < 0.001), indicating greater overall muscle fascicle dimensions. This trend was also observed in the fiber area, where working dogs had significantly larger muscle fibers than companion breeds (Figure 1B; *p* < 0.001). Additionally, the ratio of fiber area to fascicle area was notably higher in working dogs (Figure 1C; *p* < 0.05), suggesting that in these breeds, the larger fiber size contributes more substantially to the overall fascicle size compared to companion dogs. On the other hand, companion dogs displayed a significantly higher number of fibers per fascicle compared to working breeds (Figure 1D; *p* < 0.001), indicating a denser packing of muscle fibers within the fascicles in these dogs. In terms of nuclei per fiber, working dogs had a significantly greater number of nuclei per fiber than companion dogs (Figure 1E; *p* < 0.001). In terms of MYH isoform, MYH2 was distributed at a significantly higher percentage in working dogs (Figure 1F; *p* < 0.001), while companion dogs displayed a significantly higher distribution of MYH4 (Figure 1G; *p* < 0.001). MYH7 was significantly more prevalent in working dogs (Figure 1H; Figure 2; *p* < 0.001).

### 3.2. Biceps Femoris

**Figure 3:**
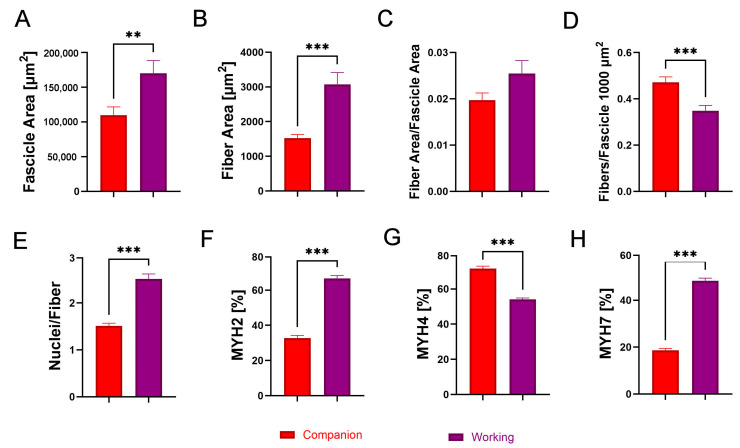
Comparative analysis of (**A**) fascicle area [µm^2^], (**B**) fiber area [µm^2^], (**C**) fiber area per fascicle area, (**D**) fibers per 1000 µm^2^ of fascicle, (**E**) nuclei per fiber, (**F**) percentage of MYH2-immunoreactive (IR) fibers, (**G**) percentage of MYH4-IR fibers, and (**H**) percentage of MYH7-IR fibers in the biceps femoris of companion (red) and working (purple) dogs. Asterisks (*) indicate significant differences between companion and working dogs (** *p* < 0.01, *** *p* < 0.001).

**Figure 4:**
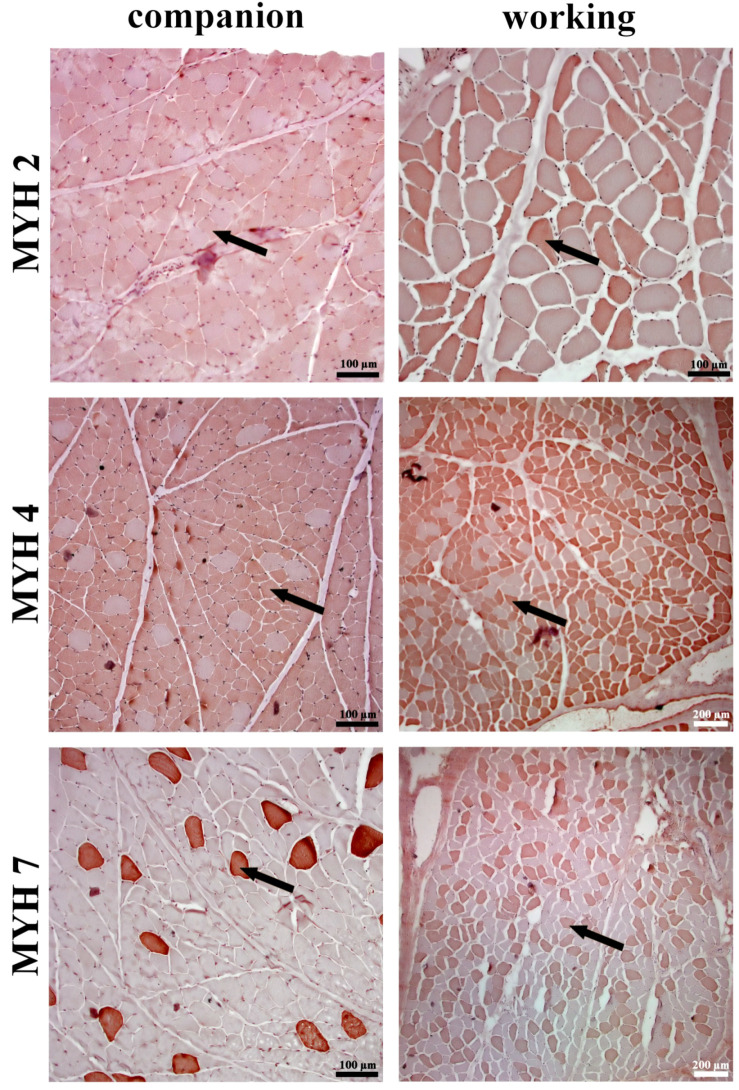
Immunohistochemical staining of muscle fibers in the biceps femoris of companion and working dog breeds, illustrating the distribution of different myosin heavy chain (MYH) isoforms. Staining was performed using DAB as a chromogen. The rows represent different myosin heavy chain isoforms, with MYH2, MYH4, and MYH7 detected in companion and working breeds. Arrows indicate immunoreactive muscle fibers displaying the respective MYH isoform. Scale bars are included in each image for reference (100 µm and 200 µm).

The results of the analysis of the biceps femoris muscle between companion and working dog breeds show significant differences in various structural parameters. Working dogs exhibited a significantly larger fascicle area compared to companion dogs (Figure 3A; *p* < 0.01). This increase in fascicle size was also reflected in the fiber area, where working dogs had significantly larger fibers than companion dogs (Figure 3B; *p* < 0.001). The fiber area-to-fascicle area ratio was not significantly different between the groups (Figure 3C). However, companion dogs demonstrated a significantly higher number of fibers per fascicle compared to working dogs (Figure 3D; *p* < 0.001). In terms of cellular components, working dogs had a significantly greater number of nuclei per fiber compared to companion dogs (Figure 3E; *p* < 0.001). Regarding MYH isoform distribution, MYH2 was distributed at a significantly higher percentage in working dogs compared to companion dogs (Figure 3F; *p* < 0.001). Conversely, MYH4 was distributed at a significantly higher percentage in companion dogs than in working dogs (Figure 3G; *p* < 0.001). Lastly, MYH7 showed a significantly higher distribution in working dogs compared to companion dogs (Figure 3H; Figure 4; *p* < 0.001).

### 3.3. Fibers Distribution

**Figure 5:**
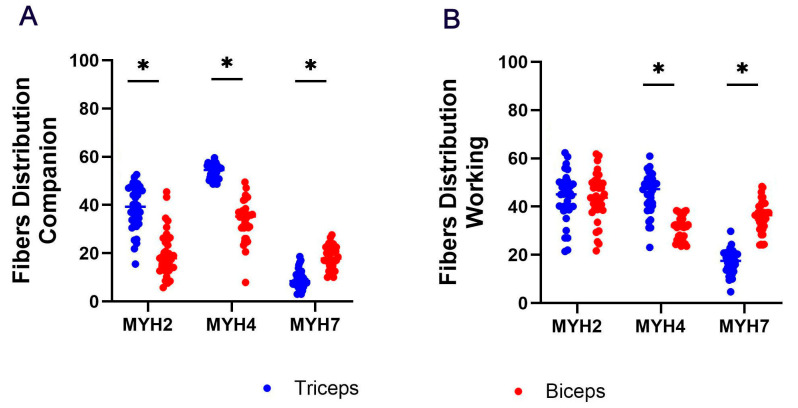
MYH2, MYH4 and MYH7 fiber distribution in triceps brachii and biceps femoris in (**A**) companion and (**B**) working dog breeds. Asterisks (*) indicate significant differences between the muscles in fiber distribution (* *p* < 0.05).

In companion dogs, the most abundant fiber type observed in both the triceps brachii and biceps femoris was MYH4, with the triceps brachii exhibiting a higher proportion of these fibers compared to the biceps femoris (*p* < 0.05). MYH2 fibers were the second most prevalent fiber type, with a greater proportion present in the triceps brachii relative to the biceps femoris (*p* < 0.05). MYH7 fibers constituted the smallest proportion of fibers, with the triceps brachii displaying a lower percentage compared to the biceps femoris (Figure 5A; *p* < 0.05). In working dogs, the proportion of MYH2 and MYH4 fibers was relatively similar overall, but MYH7 fibers were significantly less prevalent. When comparing between muscles, the distribution of MYH2 fibers was similar in both the triceps brachii and biceps femoris, while MYH4 fibers were more abundant in the triceps brachii (*p* < 0.05). However, the biceps femoris exhibited a significantly greater percentage of MYH7 fibers (Figure 5B; *p* < 0.05).

### 3.4. Companion Breeds’ Muscle Comparison

**Figure 6:**
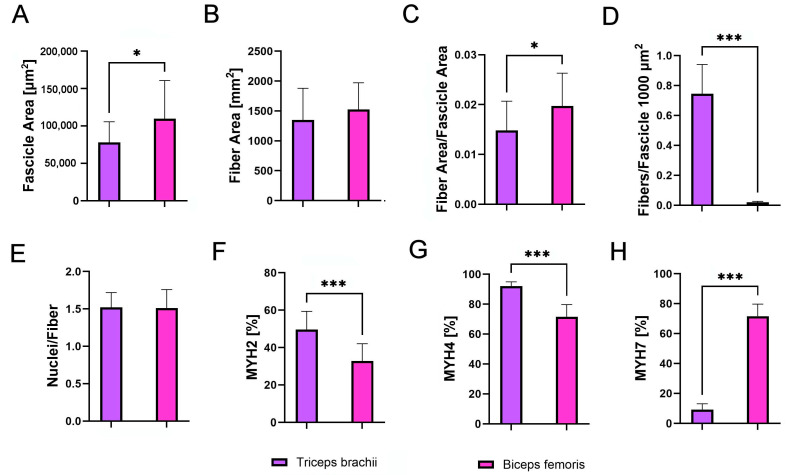
Comparative analysis of (**A**) fascicle area [µm^2^], (**B**) fiber area [µm^2^], (**C**) fiber area per fascicle area, (**D**) fibers per 1000 µm^2^ of fascicle, (**E**) nuclei per fiber, (**F**) percentage of MYH2-immunoreactive (IR) fibers, (**G**) percentage of MYH4-IR fibers, and (**H**) percentage of MYH7-IR fibers in the triceps brachii (purple) and biceps femoris (pink) of companion dogs. Asterisks (*) indicate significant differences between triceps brachii and biceps femoris (* *p* < 0.05, *** *p* < 0.001).

A comparative analysis of the triceps brachii and biceps femoris within the group of companion dogs revealed a significant difference between these muscles. The biceps femoris exhibited a larger fascicle area and a higher fiber area-to-fascicle area ratio (Figure 6A,C; *p* < 0.05, *p* < 0.05). However, no significant differences were observed between the muscles in the fiber area (Figure 6B). Regarding the number of fibers per fascicle, it was found to be significantly higher in the triceps brachii (Figure 6D, *p* < 0.001). No significant changes were noted in the number of nuclei per fiber (Figure 6E). However, significant differences were identified in the percent distribution of MYH isoforms in muscle fibers. Specifically, MYH2 and MYH4 predominated in the triceps brachii (Figure 6F,G; *p* < 0.001, *p* < 0.001), while the percentage of MYH7 was higher in the biceps femoris (Figure 6H).

### 3.5. Working Breeds’ Muscle Comparison

**Figure 7:**
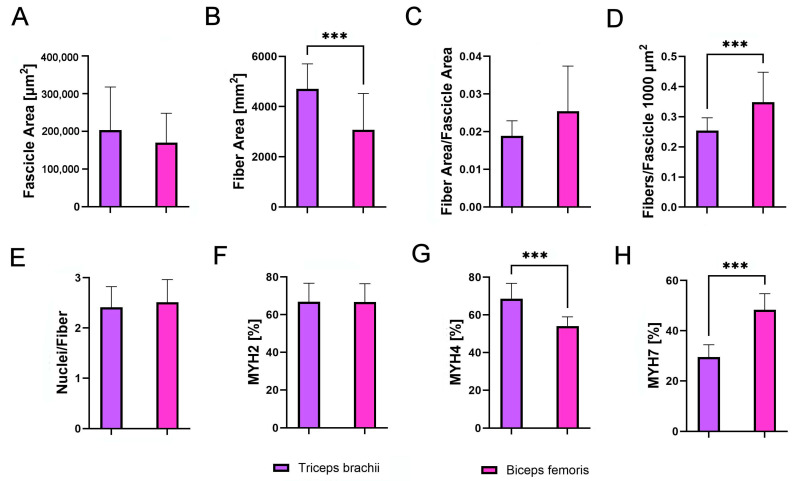
Comparative analysis of (**A**) fascicle area [µm^2^], (**B**) fiber area [µm^2^], (**C**) fiber area per fascicle area, (**D**) fibers per 1000 µm^2^ of fascicle, (**E**) nuclei per fiber, (**F**) percentage of MYH2-immunoreactive (IR) fibers, (**G**) percentage of MYH4-IR fibers, and (**H**) percentage of MYH7-IR fibers in the triceps brachii (purple) and biceps femoris (pink) of working dogs. Asterisks (*) indicate significant differences between triceps brachii and biceps femoris (*** *p* < 0.001).

A comparative analysis of the triceps brachii and biceps femoris in the group of working dogs did not reveal a significant difference in fascicle area between the muscles (Figure 7A). However, the triceps brachii exhibited significantly larger muscle fibers (Figure 7B, *p* < 0.001). There were no differences between the muscles in parameters such as the fiber area-to-fascicle area ratio (Figure 7C), the number of nuclei per fiber (Figure 7E), or the percentage of MYH2 isoform in muscle fibers (Figure 7F). Significant differences were observed in the number of fibers per fascicle, which was higher in the biceps femoris (Figure 7D, *p* < 0.001), the percentage of MYH4, which was greater in the triceps brachii (Figure 7G, *p* < 0.001), and the percentage of MYH7, which was higher in the biceps femoris (Figure 7H, *p* < 0.001).

## 4. Discussion

This study investigated the muscle fiber composition and structural adaptations in the triceps brachii and biceps femoris muscles of working and companion dog breeds. By analyzing fiber morphology, fiber area, MYH isoform distribution, and nuclei per fiber, we aimed to uncover breed-specific muscle adaptations shaped by selective breeding for physical roles. Our results showed that working dogs possess significantly larger fibers, a higher proportion of type IIa and type I fibers, and an increased number of nuclei per fiber compared to companion breeds, which exhibited a higher proportion of type IIb fibers. These findings suggest that working breeds are structurally adapted for endurance and strength, while companion dogs demonstrate muscle characteristics better suited for short bursts of activity [26]. The study expands on previous research by offering a comparison of muscle fiber composition across these two breed groups, highlighting the significant impact of selective breeding on canine muscle morphology.

Kuzon et al. [27] conducted a histochemical and morphometric analysis of various hindlimb muscles, including the gracilis, sartorius, and tibialis anterior, in mixed-breed, hound-type dogs and in Beagles. Their study found that fiber type composition remained relatively stable across dog types, with no significant differences in the proportions of type I and type II fibers between groups. This contrasts with our findings, where we observed clear distinctions in fiber type composition between working and companion dogs, and our findings are in line with the conclusions drawn by Acevedo and Rivero, who highlighted the predominance of type IIa fibers in the muscles of breeds that are usually bred for tasks requiring endurance and rapid movements [20]. Acevedo’s study particularly emphasized the functional versatility of type IIa fibers, which have both oxidative and glycolytic capacities, making them well-suited to the variable physical demands placed on working dogs, such as hunting or herding [20,28]. Similarly, our findings on the increased nuclei per fiber in working breeds suggest a heightened capacity for muscle regeneration, which may be essential for breeds that are exposed to high levels of physical activity [29,30]. This observation is consistent with Acevedo’s characterization of working breeds, which often display heightened regenerative abilities due to their muscular demands. In contrast, companion breeds, which are often smaller in size, showed fewer nuclei per muscle fiber, potentially contributing to their lower regenerative capacity. This difference in muscle fiber composition may partly explain why, despite being less prone to cranial cruciate ligament ruptures, small dogs often experience prolonged recovery times when affected [31,32,33].

Our study found a significantly higher proportion of type IIb fibers in companion dogs, a result that aligns with their less physically demanding lifestyle [34]. These type IIb fibers, characterized by their rapid contraction speed but low endurance, are suited for quick bursts of activity rather than sustained exertion. This result mirrors findings in van Boom et al. [3], who demonstrated that small dogs, such as toy breeds, typically exhibit a higher percentage of glycolytic fibers such as type IIb, correlating with their low endurance and sporadic energy expenditure. This fiber type distribution reflects a distinct physiological adaptation where companion breeds are optimized for agility or short bursts of activity, rather than prolonged, intense work [35].

The role of type I fibers (slow-twitch, oxidative) in endurance is another critical point of comparison. Our study showed a significantly higher distribution of type I fibers in working dogs compared to companion breeds, further reinforcing the idea that working dogs are more reliant on endurance muscle fibers. This aligns well with the findings of van Boom et al. [3], who reported a similar pattern in larger breeds such as the German Shepherd and Border Collie, both of which have been bred for sustained, long-duration physical tasks such as herding or tracking. Similarly, Agüera et al. [21] found that in breeds bred for endurance and strength, such as the Spanish Greyhound and German Shepherd, type I fibers were more prominent in muscles responsible for prolonged activities, highlighting their functional role in supporting endurance.

Interestingly, our results also offer insights into potential breed-specific adaptations that were less emphasized in earlier studies. For instance, while Acevedo and Rivero [20] noted the presence of hybrid fibers, our study found clear demarcations between fiber types with significantly different functional roles across the two groups. This highlights the potential for working dogs to rely heavily on specific fiber types, depending on their physical tasks, while companion breeds exhibit a more homogeneous distribution of fiber types, adapted for low-intensity, high-frequency activities such as postural maintenance and brief, rapid movements. This contrasts with some earlier work that suggests a higher proportion of hybrid fibers in canine muscle groups across the board [20], indicating that our findings may represent breed-specific functional specializations.

A key point of divergence between our findings and earlier studies lies in the fiber density per fascicle observed in companion dogs. Our study revealed a significantly higher number of fibers per fascicle in companion breeds, which suggests a greater muscle density relative to their overall muscle size. This finding contrasts with the results from Agüera et al. [21], who reported relatively uniform muscle fiber densities across breeds, with no significant differences in fiber packing density. However, this difference may be explained by the breeds selected for our study, which focused on toy and small companion breeds, such as Shih Tzu and Yorkshire Terrier. These breeds may have developed denser fiber packing as an adaptation to maintaining posture and engaging in high-frequency but low-intensity activities, consistent with findings by Guy and Snow [19], who noted that smaller breeds tend to exhibit higher fiber density in postural muscles. Additionally, it is important to acknowledge that the smaller size of companion breeds overall could also contribute to the observed differences in fascicle size, as smaller animals may inherently have smaller fascicles, which could result in the appearance of a greater fiber density. This potential influence of animal size on fascicle characteristics should be considered when interpreting the results.

The muscle fiber composition of companion and working dogs can be compared to that of other species with similar lifestyles and functional demands. Companion dogs, which often engage in short bursts of high-intensity activity, show a predominance of glycolytic fibers, especially type IIb fibers in muscles such as the biceps femoris. This is similar to thoroughbred racehorses, whose superficial gluteus medius layers are optimized for rapid, powerful movements [36,37]. Likewise, southern African wild ruminants such as kudu exhibit a high proportion of glycolytic fibers, enabling quick and powerful movements, making their muscle profile comparable to that of companion dogs [38]. In contrast, the more balanced fiber composition of working dogs, which includes both type IIa and type I fibers, supports their need for both power and endurance. This is similar to the deeper muscle layers in racehorses, which are adapted for endurance as well as strength, allowing them to sustain prolonged physical activity [36]. Additionally, working dogs share similarities with animals such as wildebeest and Blesbuck, which have a mixed muscle fiber profile suited for sustained locomotion over long distances [38]. Reindeer also display comparable adaptations, with muscles capable of both endurance and rapid contraction, reflecting the dual demands placed on working dogs during activities such as herding or hunting [39,40]. These comparisons highlight how selective pressures shape muscle composition across species to meet specific functional demands, whether for short bursts of speed in companion dogs or sustained, versatile activity in working breeds.

The differences between the triceps brachii and biceps femoris muscles in companion and working dogs reflect how selective breeding and functional demands could shape muscle structure. In quadrupeds, forelimbs are primarily responsible for braking and stabilizing movements, while hindlimbs drive propulsion during locomotion [41]. In companion dogs, the biceps femoris showed a larger fascicle area and a higher fiber area-to-fascicle area ratio compared to the triceps brachii, which may relate to its role in propulsion during the short bursts of speed typical for these breeds. The triceps brachii, with its higher fiber density per fascicle, appears better suited for tasks requiring precision and postural stability, functions that align with the companion group’s less physically demanding lifestyle. While MYH7 predominates in the biceps femoris, supporting endurance potential, other factors, such as mitochondrial density, vascular supply, and oxidative enzyme activity, may also contribute to differences in muscle function, even when the MYH isoform distribution is similar. In working dogs, the triceps brachii exhibited larger muscle fibers than the biceps femoris, suggesting greater force generation capabilities for stabilization during high-intensity activities such as herding or hunting [42]. Despite similarities in nuclei per fiber and MYH2 distribution, the structural differences between the muscles indicate functional specialization. The biceps femoris, with a higher percentage of MYH7, supports sustained activity during propulsion, while the triceps brachii adapts to manage the mechanical loads of braking and stabilizing during dynamic movement. These differences align with the working breeds’ need for endurance and energy efficiency over extended periods of physical activity.

One limitation of our study is that, due to ethical constraints prohibiting experimental procedures on dogs, we were restricted to using dogs that had died naturally in veterinary clinics. Consequently, the groups of dogs used in the study were not as uniform as would have been ideal. While we made efforts to select dogs of a similar age and background, gathering information about diet and environment through interviews with owners, this variability may have introduced some uncontrolled factors. Although earlier work by Braund et al. [43] suggested that age does not significantly affect muscle fiber type distribution in dogs, more recent research by Braga et al. [44] on German Shepherds has indicated that age may indeed influence fiber composition, particularly showing an increase in type I fibers and a decrease in type II fibers with advancing age in males. These conflicting findings highlight the potential influence of age, which, despite our efforts to standardize the groups, could have impacted our results.

## 5. Conclusions

This study provides a comprehensive comparative analysis of muscle fiber composition in the triceps brachii and biceps femoris muscles of working and companion dog breeds. Our findings confirm significant morphological and functional differences in muscle fiber types between these two groups, likely as a result of selective breeding for specific physical demands. Working breeds exhibited larger muscle fibers, a higher proportion of type IIa and type I fibers, and more nuclei per fiber, all of which are adaptations that support the endurance and strength required for physically demanding tasks such as herding or hunting. In contrast, companion breeds showed a higher proportion of type IIb fibers, which are associated with short bursts of high-intensity activity, reflective of their less physically strenuous lifestyle. The increased number of myonuclei in working breeds suggests a superior regenerative capacity in their muscles, which is crucial for recovery from the physical challenges they face. Conversely, companion breeds, particularly smaller dogs, demonstrated fewer nuclei per fiber, which may contribute to their slower recovery from injuries, such as cranial cruciate ligament ruptures, despite their lower incidence of such injuries.

## Figures and Tables

**Table 1:** Overview of the breeds included in the study, detailing their weight, age at the time of death, and sex.

Breed	Age (Years)	Weight (kg)	Sex
Companion Breeds			
Miniature Pinscher	8	5	F
Shih Tzu	7	7	M
Pekingese	9	6	M
English Bulldog	7	23	F
French Bulldog	6	14	M
Yorkshire Terrier	10	3	F
Working Breeds			
Labrador Retriever	10	27	F
Labrador Retriever	8	30	M
Golden Retriever	7	31	M
Dachshund Wirehaired	10	11	F
Border Collie	11	12	F
Jack Russel Terrier	10	7	F

**Table 2:** Primary and secondary antibodies used in the study.

Antibody	Host	Catalog Number	Dilution	Manufacturer
Primary antibody				
Anti–MYH2	rabbit	55069-1-AP	1:50	ProteinTech Group, Chicago, IL, USA
Anti–MYH4	rabbit	20140-1-AP	1:200	ProteinTech Group, Chicago, IL, USA
Anti–MYH7	rabbit	DF12122	1:200	Affinity Biosciences, Changzhou, China
Secondary antibody				
Anti-mouse/Anti-rabbit	goat	DPVB-HRP	RTU ^1^	ImmunoLogic, Duiven, The Netherlands

^1^ RTU = Ready To Use.

## Data Availability

Data are contained within the article.
